# To validate the integral conceptual model of frailty among community-dwelling older adults in China: a cross-sectional study

**DOI:** 10.1186/s12877-023-03960-9

**Published:** 2023-04-21

**Authors:** Jun-Yao Fan, Wen Xie, Wen-Ya Zhang, Yue-Ting Liu, Quan Wang, Hui-Min Zhao, Ling-Lin Kong, Jie Li

**Affiliations:** 1 Department of Nursing, Tongji Hospital, Tongji Medical College, Huazhong University of Science and Technology, Wuhan, China; 2 School of Nursing, Tongji Medical College, Huazhong University of Science and Technology, 13 Hangkong Road, Qiaokou District, Wuhan, China

**Keywords:** Accidental falls, Disease, Frail elderly, Quality of life

## Abstract

**Purpose:**

The integral conceptual model of frailty (ICFM) integrates physical, psychological, social aspects of individuals and stresses that frailty is a dynamic state evolving over time. This study aimed to validate the ICMF among community-dwelling older adults in China.

**Methods:**

The study recruited 341 older community-dwelling adults by convenient sampling method between June 1 and August 30, 2019 in Hubei province, China. The data was collected by questionnaire-based survey. Frailty was assessed by the Chinese version of the Tilburg Frailty Index. Participants were assessed for life-course determinants, disease and adverse health outcomes. Hierarchical regression analyses, Bootstrap method and the structural equation model were conducted in data analysis.

**Results:**

Both linear and logistic hierarchical regression models were statistically significant. Life-course determinants, disease, and three domains of frailty together explained 35.6% to 50.6% of the variance of disability and all domains of quality of life. The mediation effect of frailty between disease and all adverse outcomes was significant, excluding hospitalization. The structural equation model guided by the ICMF fits the data well.

**Conclusions:**

The ICMF is valid among community-dwelling older adults in China. Therefore, the multidimensional concept of frailty should be widely used in Chinese communities.

## Introduction

With the increasing aging population around the world, the challenge caused by aging greatly impacts society. China has the largest older population in the world. Data from the National Bureau of Statistics of China [1], the number of older adults over 60 in China has reached 254 million, accounting for 18.1% of the total population of China by the end of 2019. This means that China will face greater challenges brought by the aging process.

Frailty, a complex clinical syndrome, has been highly focused on in the field of gerontology [2]. Many studies show that frailty is associated with adverse health outcomes, such as falls [3], disability [4], hospitalization [5], and death [6]. Therefore, the theoretical model of frailty should be better understood by health care workers and appropriate tools should be adopted to identify the frailty of older adults. It is helpful to reduce the risk of adverse health outcomes, improve the quality of life, and finally achieve healthy aging for older adults.

In the study process of frailty, researchers have put forward many theoretical models of frailty based on their findings and cultural background, leading to different definitions and measurements of frailty [2]. Currently, two distinct models of frailty are considered for the definitions of frailty [7]: the one-dimensional predominately physical frailty, such as the Cardiovascular Health Study Phenotype Model [8]; and the multi-dimensional, predominately biopsychosocial frailty, which also integrates psychological and social, besides physical function, such as the integral conceptual model of frailty (ICMF) [9]. Up to now, Chinese researchers and health workers mainly use the one-dimensional concept of frailty [10]. However, many researchers argue that the one-dimensional definition of frailty is too partial to result in comprehensive care for older adults, neglecting the individual’s wholeness and reducing the quality of care delivered to frail older adults [11]. Therefore, it is high time to adopt the multi-dimensional definition of frailty among community-dwelling older adults in China.

Prior to the formulation of the ICMF, there were many conceptual models of frailty focusing mainly on the physical function of older adults, such as the Phenotype Model and the Cumulative Deficit Model [8, 12]. Concerned that older adults as a whole person will be neglected, Gobbens et al. proposed the ICMF in 2010 through a literature search and expert meetings [9, 11, 13]. The ICFM integrates physical, psychological, social aspects of individuals and stresses that frailty is a dynamic state evolving over time. In the ICFM, (physical, psychological, social dimensions of) frailty is affected by life-course determinants (including age, education, income, sex, ethnicity, marital status, living environment, lifestyle, life events and biological) and disease(s), that can lead to adverse health outcomes (including falls, disability, quality of life and death) [9]. The model was proposed with three hypotheses: first, life-course determinants and frailty affect adverse outcomes; second, the effect of disease on adverse outcomes is mediated by frailty. Another hypothesis considering that frailty relies on life-course determinants to affect adverse outcomes was omitted because the hypothesis was rejected in their previous research [14]. Subsequently, Gobbens et al. conducted a series of confirmatory studies on ICFM among older adults in Dutch nursing home and community, and the results indicated the importance and scientific nature of ICFM [14, 15]. In addition, a multi-dimensional tool for evaluating frailty based on the ICMF has been developed, the Tilburg Frailty Index (TFI) [16]. The reliability and validity of ITF have been tested to be good in many countries, such as German [17], Turkey [18], Spain [19], etc., and it is widely adopted to measure the multi-dimensional frailty of older adults.

Through literature review, there is a lack of confirmatory study on the ICMF in China. Only Dong et al. verified the reliability and validity of TFI among Chinese community-dwelling older adults [20], and Si et al. compared the diagnostic properties of various frailty assessment tools including TFI in Chinese community-dwelling and institutional older adults [21, 22]. Studies focused on the reliability and validity of TFI rather than the ICMF. According to the 2019 China Health Statistics Yearbook [23], medical services are mainly provided by general or specialists practitioners in hospitals and clinics in China, which is inconsistent with the Dutch healthcare services in Gobbens's study [15], and incidence of chronic disease is also different with it. Some studies have shown some difference in measurement of frailty between China and Netherlands [20–24]. So we want to verify the effectiveness of the ICMF among older adults in China on the basis of the former study, for which we can promote the proper application of this model in China.

So, the present study aims to verify the ICMF in Chinese community-dwelling older adults by testing two hypotheses of the ICFM in the same way as Gobbens et al. [14]: Hypothesis 1: life-course determinants and frailty affect adverse outcomes; Hypothesis 2: the effect of disease on adverse outcomes is mediated by frailty, and analyzing the fitting situation of data with the structural equation modeling (SEM) guiding by ICMF.

## Methods

### Study population and data collection

This is a questionnaire-based cross-sectional study. The study adopted a convenient sampling method, recruiting older adults from the community center in 4 cities of Hubei province, China: Tianmen, Huanggang, Wuhan, and Xiaogan between June 1 and August 30, 2019. Surveyors with uniform training conducted a questionnaire survey on the subjects meeting the inclusion criteria through a household survey. The inclusion criteria were the following: adults over 60; live in the community; informed consent and voluntary to participate in this investigation. Exclusion criteria were: poor listening or comprehension ability to communicate. Researchers with uniform training approached 377 eligible subjects, then introduced the purpose, significance and process of the study in detail to them one by one, 15 of whom refused. After obtaining the informed consent of the subjects who agreed to cooperate with the investigation, a one-to-one questionnaire survey was conducted, 12 subjects dropped out. Finally, 350 structured questionnaires were obtained, 9 were ultimately excluded from the analysis due to incomplete or missing data. In total, 341 older adults were included in the study. The main statistical analysis method in this study is regression analysis and SEM. In regression analysis, the sample size is generally required to be 10 times the number of independent variables [25], there are 13 independent variables, so the sample size should be at least 130. And for SEM, the sample size is recommended to be at least 200 [26]. In this study, A total of 341 old adults were included, which is sufficient for statistical analysis. All the participants underwent a questionnaire-based face-to-face survey. The study was approved by the Ethics Committee of Tongji Medical College (Approval number: S941). Before participating in the study, each participant was informed consent and told that they could withdraw from the survey at any time.

### Life-course determinants

According to the ICMF [9], age, gender, marital status, ethnicity, monthly personal income, self-reported lifestyle, level of education, life events (experienced widowhood or divorce or retired or a close relative become seriously ill within a year) and whether satisfaction with dwelling environment were inquired.

### Disease

The status of chronic diseases was recorded by asking “What chronic diseases have you been diagnosed with in the past?” The total number of recorded diseases of each participant was used for statistical analysis.

### Frailty

The total and physical, psychological, social dimensions of frailty were assessed by the Chinese version of TFI, which had been proven valid and reliable for assessing comprehensive frailty in Chinese community-dwelling older adults [20]. It consists of 15 items, of which eight items correspond to physical frailty (self-conscious health condition, weight loss, walk, poise, hearing, eyesight, strength in hand, and physical fatigue), points ranging from 0 to 8; four items correspond to psychological frailty (coping capacity, cognition, depressive and anxiety symptom), points ranging from 0 to 4; three items correspond to social frailty (living alone, social relations and support), points ranging from 0 to 3. The total points of frailty ranged from 0 to 15. A higher score indicates more frailty. In this study, the Cronbach’s alpha coefficient of TFI was 0.71.

### Adverse health outcomes

Activity of daily living (ADL) or instrumental activity of daily living (IADL) was used as indicators of functional ability. Due to the low incidence of ADL in our participants, Lawton IADL scale [24] was used to assess disability. Accordingly, IADL disability was based on whether there is any difficulty or needing help in using telephone by yourself, preparing meals by yourself, washing your own clothes, cleaning-housework, shopping for items, using public transport, taking medicine by yourself, or managing money. In each item, having difficulty or needing help scored 0, finishing independently scored 1. The total score ranged from 0 to 8.

Falls was recorded by asking “Have you fallen in a year?” with response categories “yes” or “no”. Visiting the clinic, using the emergency department and hospitalization were used to assess health care utilization. By asking “Have you been to the clinic/emergency/hospital in the past year?”, with response categories “yes” or “no”. “yes” counts 1, “no” counts 0.

The Chinese version of the Short Form 12 Health Survey Questionnaire (SF-12v2) was used to assess the quality of life. Study showed it was a reliable and valid health-related quality of life instrument for Chinese older adults [25]. The instrument had eight domains that are, Physical Functioning (PF), Role Physical (RP), Bodily Pain (BP), General Health (GH), Vitality (VT), Social Functioning (SF), Role Emotional (RE), and Mental Health (MH). In this study, standardized scores were unnecessary because there was no need to compare them with other populations. Therefore, we used the original scores for analysis. In this study, the Cronbach’s alpha coefficient of SF-12v2 was 0.85.

### Statistical analysis

The Statistical Package for Social Sciences, version 21.0 (SPSS IBM Corp) and SPSS Amos, version 21.0 were used in analyses. Linear hierarchical regression analyses and logistic hierarchical regression analyses were performed respectively with the continuous outcomes (disability and eight domains quality of life) and dichotomous outcomes (fall, visiting the clinic, using the emergency department and hospitalization) as the dependent variables. In the hierarchical regression, life-course determinants (age, education and monthly personal income [entered as continuous variables], gender, marital status, self-report lifestyle, life events and whether satisfaction with living environment [entered as dummy variables, sex: ‘1’ man, ‘0’ woman; marital status: ‘1’ married, ‘0’ rest; self-report lifestyle: ‘1’ healthy, ‘0’ rest; life event: ‘1’ happened, ‘0’ not happened; residence: ‘1’ unsatisfactory, ‘0’ satisfactory]) were entered in the first step of the regression analyses. Ethnicity was excluded in further analysis, because there were too few non-Han participants (0.3%). In the second step, the number of diseases was entered. The three domains of frailty were finally entered. The significance of each block and total regression model was tested by the F-test of linear regression, and the x^2^-test of logistic regression. The first hypothesis mentioned above was tested by significance of all regression coefficients (*B*) and *R*^*2*^ from the first and third blocks. The second hypothesis concerning the mediating effect of frailty in relation to disease and adverse outcomes was analyzed by Bootstrap method utilizing PROCESS Procedure for SPSS described by Hayes [27]. In the present analysis, we applied 5000 bootstraps. Finally, based on the ICMF and these analyses, we used the SEM to assess whether the model fit data. Factor loading reaches a significant level with the chi square degree of freedom ratio (χ^2^/df) < 3, the goodness-of-fit index (GFI) > 0.90, the incremental fit index (IFI) > 0.90, the compare fitting indices (CFI) > 0.90, and the root-mean-square error of approximation (RMSEA) < 0.08 indicated the model fit data well [28]. All statistical analyses adopted a significance level of two-sided 0.05.

## Results

### Sample characteristics

A total of 377 questionnaires were issued, and 341 valid questionnaires were collected, with the effective response rate of questionnaire was 90.5%. Mean age of participants was 68.6 years, 58.9% were female, 59.5% had no formal education or primary education level, 44.6% had less than 1000RMB monthly personal income, 61.3% reported healthy lifestyle, 60.7% had experienced life events in the past year, 87.1% were satisfied with residence, and 37.2% was identified as frail, 41.6% was IADL disability. Population characteristics are presented in Table 1.

**Table 1:** Participant characteristics (*N* = 341)

Characteristics	*n* = 341
**life-course determinants of frailty**	
Age (y), mean ± standard deviation	68.6 ± 6.2
Sex, *n* (%)	
Male	140 (41.1)
Female	201 (58.9)
Marital status, *n* (%)	
Married	251 (73.6)
Spinsterhood	3 (0.9)
Divorced	3 (0.9)
Widowed	84 (24.6)
Ethnicity	
Han	340 (99.7)
Other	1 (0.3)
Education	
No formal education	83 (24.3)
Primary	120 (35.2)
Junior	62 (18.2)
Senior	46 (13.5)
Undergraduate	29 (8.5)
Master	1 (0.3)
Monthly personal income	
≤ 1000RMB	152 (44.6)
1001-2000RMB	74 (21.7)
2001-4000RMB	58 (17.0)
> 4000RMB	57 (16.7)
Self-report lifestyle	
Healthy	209 (61.3)
Neither healthy nor unhealthy	114 (33.4)
Unhealthy	18 (5.3)
Life event	
Happened	134 (39.3)
Not happened	207 (60.7)
Residence	
Satisfactory	297 (87.1)
Unsatisfactory	44 (12.9)
**Chronic disease**	
History of chronic disease	308 (90.3)
Number of sick, mean ± standard deviation	3.2 ± 2.2
**Frailty, mean ± standard deviation**	
Total of TFI	3.9 ± 2.8
physical domain of TFI	1.5 ± 1.8
psychological domain of TFI	1.5 ± 1.2
social domain of TFI	0.9 ± 0.8
**Adverse outcomes of frailty**	
IADL problems, *n* (%)	142 (41.6)
Points of IADL	7.0 ± 1.5
History of Fall,*n*(%)	53 (15.5)
**Health care utilization**	
History of visiting clinic, *n* (%)	209 (61.3)
History of using emergency department, *n* (%)	19 (5.6)
History of hospitalization, *n* (%)	92 (27.0)
**Quality of life, mean ± standard deviation**	
PF	4.8 ± 1.5
RP	7.7 ± 2.7
BP	3.9 ± 1.3
GH	2.7 ± 1.1
VT	4.0 ± 0.9
SF	4.1 ± 1.2
RE	8.4 ± 2.2
MH	8.4 ± 1.8

*IADL* Instrumental Activities of Daily Living, *TFI* Tilburg Frailty Indicator, *PF* Physical functioning, *RP* Role participation with physical health problems (role physical), *BP* Bodily pain; GH: general health, *VT* Vitality, *SF* Social functioning, *RE* Role participation with emotional health problems (role-emotional), *MH* Mental health

### Life-course determinants and frailty affect adverse outcomes

The results of the linear and logistic hierarchical regression analyses are presented in Table 2. The total *R*^*2*^ and *X*^*2*^ (last line of table) indicated how much of variance in the dependent variable was explained by all independent variables together, and the *R*^*2*^ and *X*^*2*^ in last line of each block indicated how much of variance was explained by predictor variables in each block. After three blocks of independent variables entered the regression model, unstandardized regression coefficients (*B*) of each predictor and their significance were also shown in the table. For continuous adverse outcomes, the total *R*^*2*^ of Table 2 showed that all independent variables explained a moderate to large part of the variance of disability and quality of life (35.6% to 50.6%).

**Table 2:** Unstandardized regression coefficients (*B*) and explained variance (*R*^*2*^ or *X*^*2*^) for line and logistic hierarchical regression analysis explaining adverse outcomes

Independent variables/ Dependent variables			Disability	Quality of life								Fall	Visiting clinic	Using emergency department	Hospitalization
				PF	RP	BP	GH	VT	SF	RE	MH				
**Life-course determinants**															
age		*B*	-0.05^c^	-0.04^c^	0.08	0.08	0.05	-0.02^c^	-0.01	0.12	0.12	0.03	-0.04	-0.02	0.02
sex		*B*	0.10	0.46^c^	-0.02	0.42^c^	0.01	-0.01	0.08	-0.04	-0.04	0.84	1.01^c^	-0.43	0.28
Marital status		*B*	-0.01	-0.01	-0.01	-0.06	0.01	-0.04	-0.05	-0.06	-0.06	0.10	-0.04	0.16	-0.44
Monthly personal income		*B*	0.21^c^	-0.12	-0.13	0.10	0.02	-0.10	-0.02	0.26^b^	-0.07	-0.34	0.01	-0.66	0.21
Education		*B*	0.07	-0.06	-0.29^a^	-0.03	-0.04	-0.06	-0.02	-0.01	-0.01	0.01	0.27	0.43	0.10
Lifestyle		*B*	0.04	0.09	-0.08	-0.03	0.25^a^	0.23^b^	0.08	0.21	0.21	0.30	0.17	-0.10	0.16
Life event		*B*	-0.01	-0.52^c^	-0.06	-0.08	-0.18	-0.06	-0.13	-0.26	-0.18	0.23	-0.98^c^	-0.88	-1.28^c^
Residence		*B*	-0.01	-0.04	-0.03	0.01	0.08	-0.02	0.00	-0.02	-0.02	-0.21	0.46	-0.51	0.67
**R**^**2**^			0.20^a^	0.22^a^	0.16^a^	0.17^b^	0.17^c^	0.14^c^	0.12^a^	0.17^b^	0.13^b^				
**X**^**2**^												31.13^c^	53.10^c^	18.08*	49.11^c^
**Disease**		*B*	0.07	-0.01	-0.02	-0.07^a^	-0.08^b^	-0.02	-0.05	-0.02	-0.09^a^	-0.05	0.20^a^	0.14	0.32^c^
**R**^**2**^			0.02^b^	0.05^c^	0.08^c^	0.09^c^	0.10^c^	0.05^c^	0.07^c^	0.03^c^	0.05^c^				
**X**^**2**^												1.72	17.84^c^	5.56^a^	25.38^c^
**Frailty**															
Physical		*B*	-0.53^c^	-0.39^c^	-0.95^c^	-0.34^c^	-0.26^c^	-0.17^c^	-0.28^c^	-0.58^c^	-0.79^c^	0.35^b^	0.35^b^	0.31^a^	0.11
Psychological		*B*	-0.05	-0.09	0.07	-0.02	-0.10^a^	-0.22^c^	-0.18^c^	-0.32^c^	-0.19^c^	0.07	-0.01	-0.12	-0.02
Social		*B*	0.00	0.04	-0.29^a^	0.04	-0.02	-0.07	-0.01	-0.07	-0.21^a^	0.17	-0.22	0.39	-0.06
***R***^***2***^			0.25^c^	0.16^c^	0.27^a^	0.15^c^	0.13^a^	0.17^c^	0.17^c^	0.19^c^	0.28^a^				
***X***^***2***^												13.67^b^	13.48^b^	5.62	1.63
**Total *R***^***2***^			0.47^c^	0.42^c^	0.51^c^	0.41^c^	0.40^c^	0.36^c^	0.37^c^	0.38^c^	0.48^c^				
**Total *X***^***2***^												46.52^c^	84.42^c^	29.34^b^	76.11^c^

*PF* Physical functioning, *RP* Role participation with physical health problems (role physical), *BP* Bodily pain, *GH* General health, *VT* Vitality, *SF* Social functioning, *RE* Role participation with emotional health problems (role-emotional), *MH* Mental health

^a^*P* < .05, ^b^*P* < .01, ^c^*P* < .001

In the first block *R*^*2*^ showed that life-course determinants explained a significant part of the variance of all adverse health outcomes. However, the significance of *B* indicated a few life-course determinants were significant, after controlling for other two types of independent variables. Older people showed IADL disability more and scored a low quality of life on PF and VT; men scored higher quality of life on PF and BP domain but visited clinic more; higher income is associated with less disability and high quality of life on RE; older adults with a healthy lifestyle had a better quality of life on GH and VT; older adults with high levels of education had a better quality of life on PR; older adults who have experienced any life events (widowhood, divorce, retired, a close relative become seriously ill) within a year visited clinic and hospitalizations more often and had a lower quality of life on PF. No effects of marital status and satisfaction of residence were found.

The *B* in the third block of Table 2 indicated that three domains of frailty explained a significant part of the variance of disability and quality of life after controlling for other two types of independent variables. Separating frailty into its domains, the effects of physical domain on disability and quality of life; psychological domain on GH, VT, SF, RE and MH domains of quality of life; and social domain on RP and MH domains of quality of life, were significant controlling for life-course determinants and disease. For dichotomous adverse outcomes, the total *X*^*2*^ (last line) in Table 2 showed that independent variables had a significant effect on four adverse outcomes. Similar to linear hierarchical regression, only a few effects of life-course determinants were significant, after controlling for other two types of independent variables. The *X*^*2*^ in the third block suggested that there was no effect of frailty on using emergency department and hospitalization. However, the *B* indicated the effects of physical frailty on dichotomous dependent variables were found excluding hospitalization, after controlling for other two types of independent variables.

### The effect of disease on adverse outcomes is mediated by frailty

As shown in Table 3, the direct effect of disease on 7 adverse outcomes was significant after further considering frailty, but 6 adverse outcomes were not significant. The mediation effect (indirect effect) of frailty between disease and all adverse outcomes was significant, excluding hospitalization, with indirect effect not including zero in the 95% CI.

**Table 3:** The effect of frailty (M) in the association between diseases (X) and adverse outcomes (Y)

Adverse outcomes	X → Y Direct effect			Indirect effect	
	Effect	SE	*P*	Effect	*95%CI*
Disability	-0.02	0.04	0.63	-0.14	[-0.28, -0.16]
Falling	0.04	0.08	0.64	0.19	[ 0.11, 0.28]
Visiting clinic	0.24	0.07	< 0.01	0.11	[ 0.04, 0.18]
Using emergency treatment	0.18	0.11	0.11	0.16	[ 0.00, 0.31]
Hospitalization	0.33	0.07	< 0.01	0.05	[-0.03, 0.13]
PF	-0.09	0.04	0.02	-0.18	[-0.23, -0.13]
RP	-0.16	0.06	0.01	-0.37	[-0.46, -0.29]
BP	-0.13	0.03	< 0.01	-0.14	[-0.18, -0.10]
GH	-0.11	0.03	< 0.01	-0.13	[-0.16, -0.10]
VT	-0.02	0.02	0.37	-0.13	[-0.16, -0.10]
SF	-0.07	0.03	0.02	-0.15	[-0.19, -0.11]
RE	-0.18	0.05	0.73	-0.31	[-0.39, -0.24]
MH	-0.03	0.04	0.55	-0.28	[-0.34, -0.21]

All analyses were performed separately according to each adverse outcome

*PF* Physical functioning, *RP* Role participation with physical health problems (role physical), *BP* Bodily pain, *GH* General health, *VT* Vitality, *SF* Social functioning, *RE* Role participation with emotional health problems (role-emotional), *MH* Mental health

### The fitting situation of data with the SEM guiding by ICMF

**Fig. 1:**
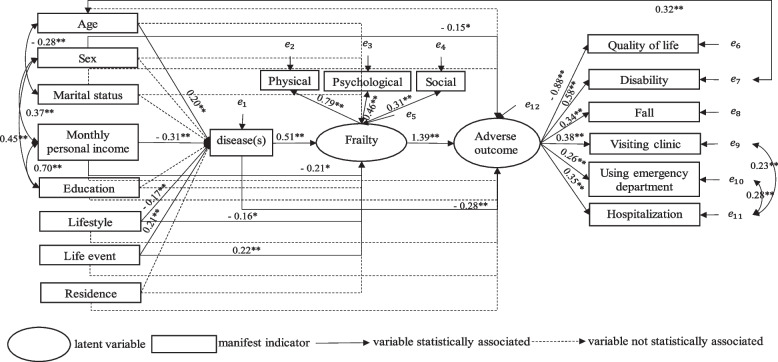
The estimated path coefficients of the model guiding by the integral conceptual model of frailty. Statistically significant standardized coefficients are included in the graph. Model fit indicators: the chi square degree of freedom ratio (χ2/df) = 2.508; the goodness-of-fit index (GFI) = 0.920; the incremental fit index (IFI) = 0.901; the confirmatory fit index (CFI) = 0.898; the root-mean- square error of approximation (RMSEA) = 0.067, *:*P* < 0.05; **: *P* < 0.001

The SEM was constructed guided by the ICMF. The statistically significant standardized coefficients are shown in Fig. 1. We performed a model modification based on the results of the model, regression analysis results and literature review, χ2/df = 2.508, GFI = 0.920, IFI = 0.901, CFI = 0.898, RMSEA = 0.067, although CFI is slightly below 0.9, other indicators all meet the criteria, indicating the SEM guiding by the ICMF fits the data well. Disease(s) is the mediating variable between life-course determinants, including age, monthly personal income, self-reported lifestyle, life events and frailty. Life-course determinants, including monthly personal income, self-reported lifestyle and life events affect adverse outcomes indirectly by frailty. Disease(s) and frailty are mediating variables between life-course determinants and frailty, disease(s) and adverse health outcomes, respectively. In the SEM, age is related to marital status and disability, income is related to education and gender, and hospitalization is related to emergency and outpatient care.

## Discussion

The results were concordant with Hypothesis 1: life-course determinants and frailty affect adverse outcomes and Hypothesis 2: the effect of disease on adverse outcomes is mediated by frailty, and the SEM guiding by ICMF fits data well, indicating that the ICMF is valid for Chinese community-dwelling older adults. This study provides new evidence for the application of the multidimensional definition of frailty in Chinese older population. At the same time, it also suggests multidisciplinary integrated care should be applied to older community-dwelling people in China.

We found many life-course determinants were influential in adverse outcomes controlled for disease and frailty. Age, gender, income level, education degree, lifestyle and life events were related to the quality of life of older adults. The results of this study are basically consistent with the previous results. Zhao concluded that the factors affecting the quality of life of older adults also included these factors through comprehensive analysis [29]. Older adults were more likely to be IADL disabled. Connolly found that age is the main factor associated with difficulty in IADL [30]. Study showed life events predicted frailty and might increase health care utilization [31]. But many effects of life-course determinants on adverse outcomes were negated after controlling for three domains of frailty and disease, indicating that disease and frailty are intermediate variables between life-course determinants and adverse outcomes.

The effects of frailty on all recorded adverse outcomes were significant controlling for life-course determinants and disease. That means people who gets higher points assessed by TFI is more often disabled, more likely to fall, has a lower quality of life, and uses health care utilization more frequently. Many previous studies also reported that frailty was associated with these adverse outcomes [31, 32]. Additionally, the effects of physical, psychological and social domains of frailty on adverse outcomes were different. Physical frailty could predict all adverse outcomes, psychological and social frailty was associated with quality of life, even after controlling for physical frailty. That emphasizes the essence of the multidimensional definition of frailty among community-dwelling older adults in China. Our above results are similar to Gobbens among community-dwelling older adults in Dutch [15].

For the second hypothesis: the effect of disease on adverse outcomes is mediated by frailty. In this part, Bootstrap method was used to assess the indirect effect of frailty on relationship between disease and adverse outcomes. Our results showed frailty mediated most the effect of disease on adverse outcomes, except for hospitalization. It proved that the hypothesis was tenable. Ma et al. clearly pointed out that chronic diseases not only affect the frailty of the elderly in the community, but also increase the risk of frailty with the increase of the number of chronic diseases [33]. Vermeiren et al. verified that frailty increased the likelihood for developing adverse health outcomes through the systematic review and meta-analysis [34]. And the mediation was complete in disability, falls, using emergency treatment and VT, RE, MH domains of quality of life. The direct effects of disease on visiting clinic, hospitalization and PF, RP, BP, GH, SF domains of quality of life should be considered.

In addition, we verified the ICMF by constructing SEM for the first time. The SEM established by the ICMF fitted well with the data of the Chinese community-dwelling older adults, which also proved that the ICMF was applicable in China. Figure 1 shows a high correlation between monthly personal income and education level. Many studies have reported the correlation between income and education in China [35, 36]. It is recommended to delete education level in the ICMF in China.

This study validated the ICMF in Chinese older adults. First, it is conducive to the application of the ICMF, and promotes research on multi-dimensional frailty in China. Second, this study provides a theoretical basis for the application of TFI. Sutton identified 38 multi-component frailty assessment tools, and concluded that TFI was the most reliable and valid one [37]. Frailty is a better indicator of health risk in older adults than biological age, and the TFI can be used to identify multiple frail people effectively. Third, the ICMF emphasizes that frailty should be evaluated from three domains of physiological, psychological and social, which is consistent with the healthy aging advocated by China and the concept of health. The comprehensive care for older adults to delay or avoid adverse health outcomes in older adults is further illustrated, which requires that elderly health-care providers acquire physical, psychological and social knowledge and skills.

This study has a few limitations that should be addressed in the future. First, we used a convenient sampling approach to recruit participants because of human and material resources limitations. It may affect the generalization of the research results. The cross-sectional design is another limitation of our study. The participants' life-course determinants, disease, domains of frailty and adverse outcomes were evaluated at the same time. Causal relationships between variables cannot be explained by this method. And death was not included in the study as an adverse outcome. Therefore, longitudinal design is needed for further study.

## Conclusion

Our study found adverse outcomes were affected by life-course determinants and frailty, and frailty is the intermediate variable between the effect of disease on adverse outcomes. And the SEM established by the ICMF fitted well with the data of the Chinese community-dwelling older adults. These indicated the integral conceptual model of frailty is applicable among community-dwelling older adults in China. We recommend the multidimensional concept of frailty should be widely used in Chinese communities.

## Data Availability

The datasets used and analysed during the current study are available from the corresponding author on reasonable request.

## Abbreviations

ICFM: Integral conceptual model of frailty
TFI: Tilburg Frailty Index
SEM: Structural equation modeling
ADL: Activity of daily living
IADL: Instrumental activity of daily living
SF-12v2: Short Form 12 Health Survey Questionnaire
PF: Physical Functioning
RP: Role Physical
BP: Bodily Pain
GH: General Health
VT: Vitality
SF: Social Functioning
RE: Role Emotional
MH: Mental Health
SPSS: Statistical Package for Social Sciences
B: Regression coefficients
GFI: Goodness-of-fit index
IFI: Incremental fit index
CFI: Compare fitting indices
RMSEA: Root-mean-square error of approximation
